# easyClock: a user-friendly desktop application for circadian rhythm analysis and visualization

**DOI:** 10.1186/s12859-025-06340-9

**Published:** 2025-12-05

**Authors:** Binbin Wu, William W. Ja

**Affiliations:** Department of Neuroscience, The Herbert Wertheim UF Scripps Institute for Biomedical Innovation and Technology, Jupiter, FL 33458 USA

**Keywords:** Rhythm detection, Open-source, Noncoding analysis tool, Circadian rhythms, Visualization

## Abstract

Circadian rhythms regulate a wide range of biological processes, and their precise characterization is essential for understanding behavioral and physiological fluctuations. However, existing tools to analyze circadian data often require coding expertise or rely on specific data acquisition software, limiting their general applicability. Here, we present easyClock, an intuitive and interactive application designed to streamline circadian rhythm analysis and visualization. The easyClock application enables simultaneous processing of multiple files, allowing users to batch-analyze and visualize diverse sets of time series data. To enhance data analysis efficiency and provide comparable results, this application integrates comprehensive methods for handling data with various waveforms and noises. Additionally, easyClock can assess inter-individual variability and group differences using linear mixed-effects modeling. All statistical results and graphs are easily viewed and exported for any selected range of data. As a demonstration, we present a re-analysis of a time-series transcriptomic dataset, highlighting the value of easyClock as an accessible, open-source tool. This easy-to-use application requires no programming expertise and can be directly installed on Windows and macOS machines in a single step.

**Supplementary Information:**

The online version contains supplementary material available at 10.1186/s12859-025-06340-9.

## Background

The circadian clock governs many physiological processes and animal behaviors, ranging from gene expression to activity, sleep, and feeding [1]. Various tools are available to acquire circadian data, such as behavioral monitors [2–6], as well as omics methods including transcriptomics, proteomics, and metabolomics [7–9]. Numerous analysis methods can be employed to determine the rhythmicity of these data, including JTK_CYCLE [10], Cosinor analysis [11], or RAIN [12]. However, most of these tools require some coding expertise [10–15]. Other graphical user interface (GUI) analyzers, such as ClockLab [16] and ShinyR-DAM [17], are either not freely accessible to users or optimized for specific behaviors. Web-based analysis solutions reduce operational difficulties but pose the risk of inadequate maintenance or computational constraints. While recent circadian rhythm analyzers tend to simplify coding operations and optimize GUI design [18], a more efficient and user-friendly tool would facilitate analysis of data collected from high-throughput experiments. To overcome operational barriers and enhance analysis efficiency, a GUI application named “easyClock” was developed to facilitate the local processing of multiple time series sets. The free-to-use easyClock application offers an intuitive interface and efficient, comprehensive mathematical methods, thereby improving user experience in data analysis and presentation within the field of chronobiology.

## Results

### Data input and preview

**Fig. 1:**
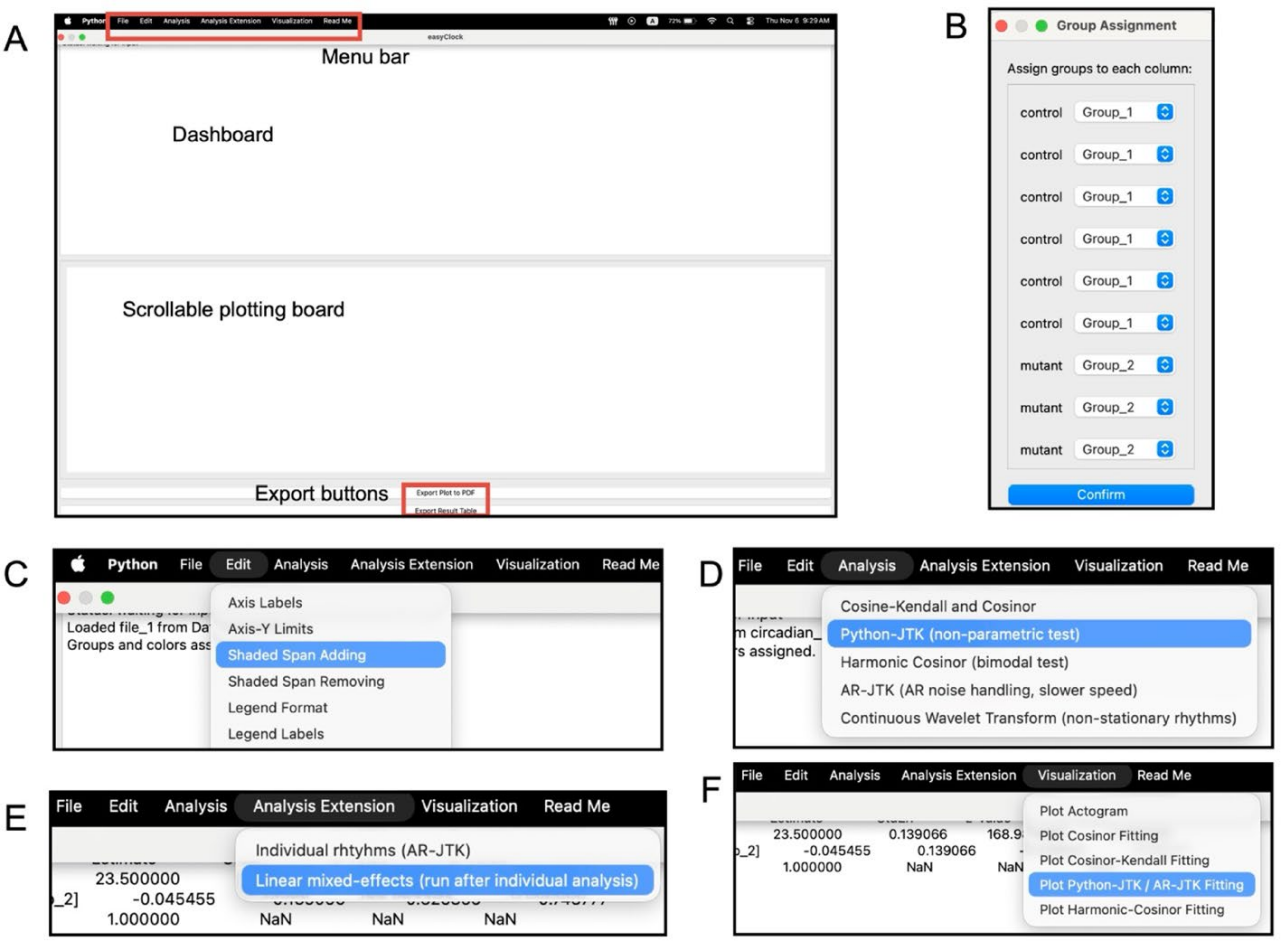
Interface of the easyClock application. **A** The easyClock interface is comprised of four distinct components: a menu bar, a dashboard, a scrollable plotting board, and export buttons. The menu bar, situated at the top of the interface, comprises menus designed to facilitate file input (**A**, **B**), plot editing (**C**), data analysis (**D**, **E**), and visualization (**F**) functionalities. Below the menu bar, the dashboard presents a comprehensive display of all execution information and analysis outcomes. The scrollable plotting board serves as a visual representation of the data, offering a preview of all datasets and enabling real-time refreshes through edit capabilities. At the bottom, two buttons are positioned to facilitate the export of plots and analysis results displayed on the boards. The analysis methods typically report four key parameters, including adjusted *p* values (ADJ.P or BH.Q), period (PER), amplitude (AMP), and acrophase (peak time)

The easyClock package applies user-centric design principles, offering a streamlined, one-step installation process. Its clean, intuitive interface is organized into modular panels that guide users sequentially through each stage, beginning with data input (Fig. 1A). As a lightweight tool, easyClock focuses on downstream analysis and visualization after basic pre-processing, rather than incorporating parsing pipelines for diverse data acquisition systems, ensuring adaptability across various experimental setups.

**Fig. 2:**
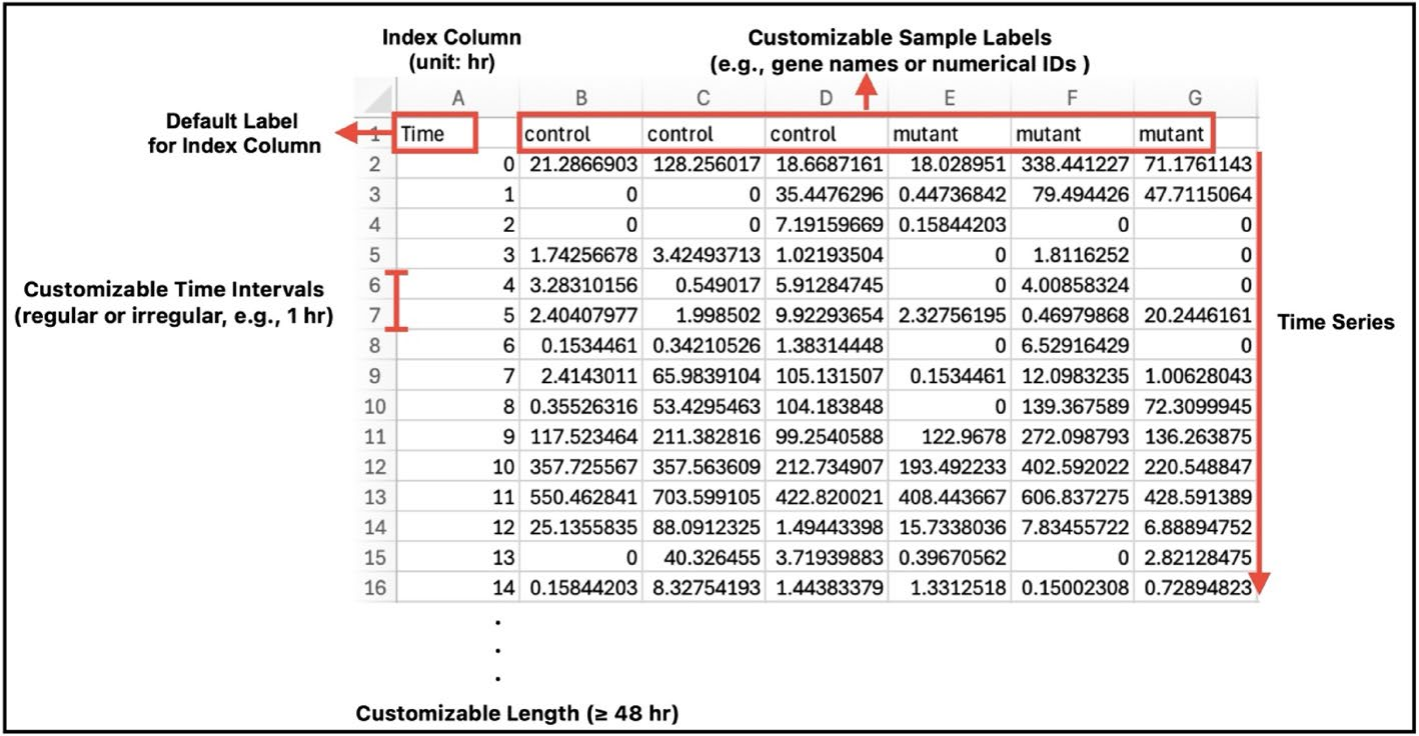
Example of input data format. The easyClock application allows users to load (or cancel) up to three CSV files, each occupying a separate slot. Each file must include an index column with a default label (“Time”), sample labels, and time-series data. The application supports both regular and irregular time intervals. Sample labels should be placed in the first row, starting from the second column, and can be customized as needed. Upon loading, group assignments are automatically generated from the first input file by grouping samples with identical labels; these groupings can be manually adjusted. When analyzing multiple files, groups in subsequent files are assigned automatically based on label matches with the first file. For robust and accurate analyses, at least 48 h of data is recommended, although there is no minimum requirement for data resolution (i.e., the number of data points per sample within a given period)

**Fig. 3:**
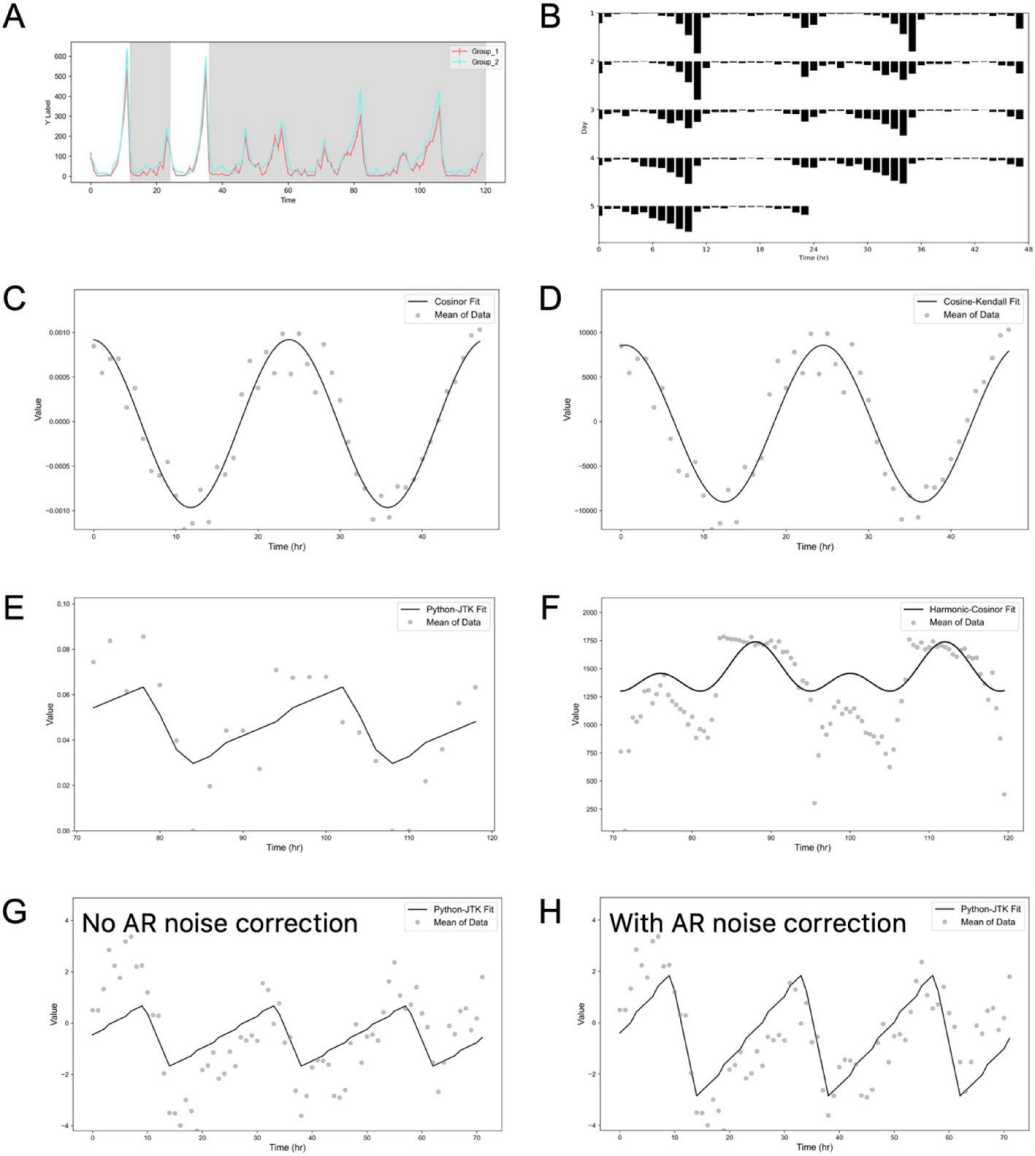
Diverse visualizations in easyClock. easyClock supports the export of various graph types, including **A** traditional time-series curves, **B** actograms, and **C–H** the best model fits. Representative examples of model fits include: **C** Cosinor, **D** Cosinor–Kendall, **E** Python-JTK, and **F** Harmonic-Cosinor. Panels **G** and **H** illustrate the effect of the autoregressive noise handling module, shown without **G** and with **H** AR noise correction

Users can load up to three CSV files simultaneously, each with user-defined time intervals (Fig. 2). When files are loaded, time series with matching identifiers (column labels) are automatically grouped. The identifiers from the first file serve as a reference; any unmatched identifiers in later files are ignored. This feature is especially valuable for comparing different phenotypes from the same experimental groups, even when their sampling intervals differ. After loading, an initial dialog box appears, allowing users to manually adjust groupings and assign colors to customize their appearance (Fig. 1B, 2). The “Edit” menu (Fig. 1C) enables an interactive preview of all datasets as conventional time-series plots. This menu also offers a shaded-span option, enabling users to highlight specific data ranges with background colors to indicate light/dark periods or other experimental conditions (Fig. 3A). Previews can be exported as publication-quality figures and are also used to guide method selection and refine parameter estimates for subsequent statistical analysis.

### Circadian rhythm analysis

Following data input and preview, users can select a file for analysis (Fig. 1D & E). All time-series within the selected file are analyzed, with users prompted to specify the period of interest; a minimum of 48 h is recommended. The current version of easyClock includes four analytical regression methods: Cosinor, Cosine-Kendall, Python-JTK, and Harmonic-Cosinor. To handle autoregressive (AR) noise [19, 20] or non-stationary oscillations [21] often present in real biological data, easyClock also integrates modules that correct for or characterize these effects. Besides, inter-individual variability and group differences can be efficiently assessed using linear mixed-effects modeling [22].

Both the Cosinor and Cosine-Kendall are designed for analyzing cosine-like rhythmic data. In easyClock, the Cosinor method applies ordinary least squares regression to fit a linearized cosine model to the data [11]: $y = M + \beta_{{1}} {\mathrm{cos}}\left( {wx} \right) \, + \, \beta_{{2}} {\mathrm{sin}}\left( {wx} \right) + \varepsilon$. Rhythmicity is assessed using an *F*-test for the null hypothesis: β_1_ = β_2_ = 0. To control for false positives across multiple comparisons within each time series (i.e., across different periods), *p*-values are adjusted using the Bonferroni correction. The Cosine-Kendall method extends the Cosinor approach by ranking the data to reduce the influence of outliers or non-Gaussian noises. It measures concordance between the ranked input and each ranked cosine reference using the non-parametric test–Kendall’s tau rank correlation [23]. The lowest *p* value from the best-fitting model is selected, and Bonferroni correction is applied for multiple period and lag tests within each time series.

The easyClock package also features a non-parametric test, Python-JTK, which is a modified reimplementation of the original JTK_CYCLE algorithm. Originally developed for R, JTK_CYCLE is widely used for detecting circadian rhythms [10]. It operates by performing rank-based comparisons between the input data and a library of discrete, triangle-shaped templates that may be either symmetric or asymmetric. In easyClock, Python-JTK uses Kendall’s tau both to measure similarity and assess statistical significance, bypassing the Jonckheere–Terpstra (JT) test employed in the original algorithm. A key advantage of Python-JTK in easyClock is its ability to automatically accommodate both regular and irregular time intervals, making it suitable for a wide range of experimental designs. To improve computational efficiency, easyClock allows users to customize parameters such as expected period, acrophase, and asymmetry, thereby reducing the number of template comparisons and accelerating the analysis. The Benjamini–Hochberg procedure is applied to adjust raw *p*-values (reported as BH.Q) when group sizes are large, while the Bonferroni correction is applied within each time series when analyzing smaller group sizes (reported as ADJ.P). To handle the AR noise commonly present in real biological data, easyClock also integrates an AR-JTK module that automatically detects and removes this noise before re-analysis.

*Drosophila* behaviors, such as sleep and locomotor activity, frequently display bimodal patterns under 12-/12-h light/dark cycles [24]. Traditional unimodal analysis methods are inadequate for capturing these complex rhythms. To address this, easyClock incorporates Harmonic-Cosinor analysis (also known as multiple-component cosinor [25]), which enables the fitting of bimodal waveforms by combining multiple sine and cosine components within each cycle (two components in the current version). The concordance between input data and each composite waveform reference is assessed using Kendall’s tau rank correlation, as demonstrated in the Cosinor-Kendall and Python-JTK methods.

Circadian rhythm data often display considerable variation in both oscillation amplitude and waveform shape. To evaluate the effectiveness of the four analysis methods in handling diverse data types, we simulated a series of arrhythmic and rhythmic datasets using Python (Sub-Table 1). Among 50 groups of pseudo-randomly generated time series, each method controlled the false positive rate (FPR) below 5% (Sub-Table 2).

**Table 1:** Comparison of rhythmicity detection methods in easyClock

Data types	Rhythmicity	Groups	Period	Amplitude	Nose (amp%)	Asymmetry
Sub-Table 1: Data simulation						
Pseudo-random	NO	50	–	–	–	–
Cosine	YES	80	24	0.001–10000	20–40%	NO
Triangle	YES	40	24	0.015–10000	30–40%	YES
Bimodal	YES	32	24	0.015–10000	20%	YES

Methods	Pseudo-random	Cosine	Triangle	Bimodal
Sub-Table 2: Data detection				
Cosinor	50/50	80/80	High FNR	31/32
Cosine-Kendall	50/50	80/80	High FNR	High FNR
Python-JTK	49/50	80/80	40/40	32/32
Harmonic-Cosinor	49/50	80/80	High FNR	32/32

Methods	FPR	Cosine	Triangle	Bimodal
Sub-Table 3: Period deviation and method recommendation				
Cosinor	0/50	0–1.5☆☆☆	–	1–3.5
Cosine-Kendall	0/50	0–2.0☆☆	–	–
Python-JTK	1/50	0–1.0☆☆	0–2.0☆☆☆	0–2.0☆☆
Harmonic-Cosinor	1/50	0–3.5☆	–	0–0.5☆☆☆

To evaluate the performance of the analysis methods in easyClock, various types of 48-h time series were simulated using Python (Sub-Table 1). Arrhythmic datasets (50 groups) were generated pseudo-randomly, while rhythmic datasets featured cosinor, triangular, or bimodal waveforms with varying amplitudes (amp) and asymmetries. Additional pseudo-random noise was added to the data as a percentage of amplitude. Four analysis methods were applied to detect rhythmicity in these datasets (Sub-Table 2). Each fraction represents the proportion of groups correctly identified as arrhythmic (in pseudo-random data) or rhythmic (in circadian datasets) by each method. “High FNR” indicates a high false negative rate (> 5%) for a given method when analyzing incompatible data types. The methods displayed differing levels of effectiveness depending on the waveform type (Sub-Table 3). The false positive rate (FPR) was calculated based on results from Sub-Table 2. Period deviation is reported as a range (e.g., 0-1.5), reflecting the extent to which the estimated period differed from the expected value across tested groups. Recommendation symbols (☆ to ☆☆☆) indicate increasing levels of suitability based on each method’s ability to detect periods accurately and minimize FPR.

To further assess method performance, we simulated various types of circadian rhythm data—including single-component cosine, asymmetric triangle waves, and bimodal patterns (Sub-Table 1). All three unimodal methods excelled at detecting cosine waveforms, even under conditions of low amplitude and high noise (Sub-Table 2 & 3). For asymmetric triangle waves, Python-JTK outperformed the other methods, especially with highly asymmetric signals (Sub-Table 2 & 3). In the case of bimodal data, Harmonic-Cosinor provided superior accuracy in period detection compared to unimodal approaches (Sub-Table 3).

**Table 2:** A comparative overview of easyClock and other alternative GUI tools

Feature	easyClock	BioDare2	pyBOAT
Platform	Standalone desktop app (macOS and Windows)	Web-based online service	Python package with GUI
Primary focus	Circadian rhythm detection	Circadian rhythm detection	Time–frequency analysis of non-stationary oscillations
Accessibility	No limitations; Runs locally	Requires registration; Runs online	No limitations; Runs locally
Open source	Yes	No	Yes
Computational capacity	Scales with local PC performance	Limited by web server capacity	Scales with local PC performance
Data privacy	Local data storage; Full privacy	Require data upload to cloud server	Local data storage; Full privacy
Key analytical methods	Harmonic Cosinor, JTK, CWT	JTK, periodogram, Detrending models	CWT, Ridge analysis
Group comparisons	Yes, includes LME in v3.0	Limited to statistical aggregation	Limited to statistical aggregation
Data input	Supports multiple files and automatic grouping (customizable by user)	Single file per dataset; grouping must be done manually	Accepts multiple single time series for independent analysis
Visualization	Editable and publication-ready plots	Non-editable	Non-editable within GUI
Output	Summary tables; Individual statistics; Circadian rhythmicity; Graphs	Summary tables; Individual statistics; Circadian rhythmicity; Graphs	Summary tables; Individual statistics; Graphs

### Visualization

In addition to data preview (Fig. 3A), easyClock enables automatic generation of various graphs (Fig. 1F). Actograms are widely used in circadian rhythm research for visualizing rhythmic patterns and phase shifts in time-series data. Users can quickly export double-plotted actograms (Fig. 3B) for any selected group with a few intuitive clicks, without the need to adjust parameters.

Furthermore, easyClock provides visualization of fitted models following analysis. For each dataset, it displays the best fit of the selected model on the original or group-averaged time series, allowing users to visually assess the quality of rhythmicity detection. Depending on the selected method, users can visualize the fitting results of Cosinor analysis, Cosine-Kendall, Python-JTK, and Harmonic-Cosinor (Fig. 3C–F). Additionally, easyClock can provide visualization of the optimal Python-JTK fit with AR correction (Fig. 3G, H).

### easyClock analysis of a transcriptomic dataset

Omics approaches are widely used to screen for cycling genes or proteins, but their scale and complexity pose challenges for rhythmicity analysis. For example, a time-series transcriptome in *Drosophila* typically comprises thousands of genes, leading to millions of statistical comparisons. To demonstrate how easyClock simplifies these procedures, we re-analyzed a previously published transcriptomic dataset.

**Fig. 4:**
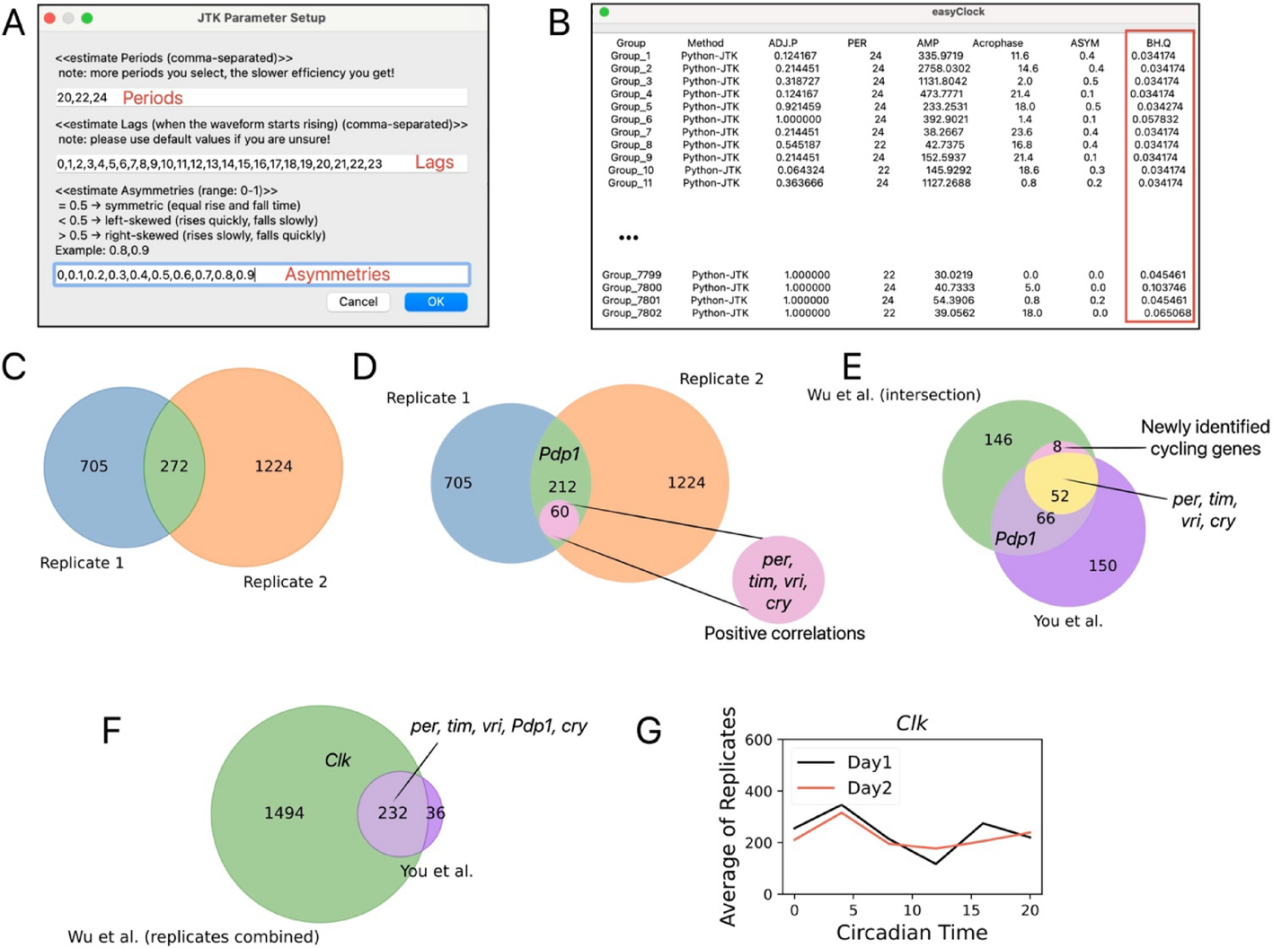
easyClock analysis of a *Drosophila* transcriptome. **A** Key parameters for Python-JTK circadian analysis. **B** Dashboard summary of Python-JTK outputs for 7,802 genes per replicate, with Benjamini–Hochberg-adjusted *p* values (BH.Q) highlighted. **C** Venn diagram showing genes with BH.Q < 0.05. The overlap represents 272 genes that cycle in both replicates. **D** Venn diagram highlighting 60 positively correlating genes within the 272-gene overlapping set. **E** Overlap between easyClock cycling genes (272 from both replicates) and previously reported cycling genes from You et al. (268 genes) [7]. Of these, 52 of the 60 positively correlated genes between replicates were also previously identified as cyclic [7].An additional 8 cycling genes, including *lolal*, were newly identified with the hybrid approach (intersection + correlation). **F** Overlap between genes identified as cyclic by grouped-replicate analysis in easyClock (1,726 genes) and You et al. (268 genes) [7], with 232 genes overlapping. **G** Relative expression levels of *Clk* across two days using the averaged values of replicates. All expression data are from the transcriptomic dataset of You et al. [7]

This transcriptome consists of 7,802 overlapping protein-coding genes across two replicates, each spanning two days with six time points per day [7]. We analyzed each replicate independently using easyClock, which required ~ 10 min for data loading and automated grouping, followed by an additional 3–5 min for dataset preview (which can be skipped) on an Apple M2-chip MacBook with 16 GB memory. Given the size and heterogeneity of the dataset, we employed the non-parametric Python-JTK test to detect cycling genes. To balance the false discovery rate (FDR) control with statistical power, raw *p* values were adjusted using the Benjamini–Hochberg procedure (BH.Q) rather than the more conservative Bonferroni correction (ADJ.P). Python-JTK enables customization of detection parameters; here, we set periods of 20, 22, and 24 h, matching the time-series resolution, and configured lags and asymmetries to capture a broad range of waveforms (Fig. 4A). Each replicate required ~ 15 min for analysis. The final output reported BH.Q, which is optimized for large group sizes, whereas ADJ.P is intended for smaller datasets (Fig. 4B).

We identified 272 genes with BH.Q < 0.05 in both replicates, including core clock genes such as *per*, *tim*, *vri*, *Pdp1*, and *cry* [26, 27] (Fig. 4C). This approach may increase the FDR since replicate-specific noise (e.g., phase-inverted fluctuations) could meet the significance threshold in both replicates without representing reproducible cycling patterns. To address this, we subsequently determined Spearman correlations between replicates for these 272 candidate genes. Sixty genes showed significant positive correlations between replicates (*p* < 0.05, *r* > 0), including *per*, *tim*, *vri*, and *cry* (Fig. 4D). Of these, 52 genes were previously reported as cyclic [7], while 8 genes—including *lolal, CG3940, CdsA, alpha-Est10, Ost48, Dh44-R2, CG11241, CG30493*—were newly identified and displayed weak cycling patterns (Fig. 4E; Supplementary Fig. 1).

Previous studies combined the replicates for analysis [7], differing from our hybrid approach (intersection + correlation). Nonetheless, easyClock supports both independent and grouped replicate workflows; grouping reduces analysis time but increases file loading duration. Using grouped replicates in easyClock, we identified 1,726 genes with BH.Q < 0.05. Among the 268 previously recognized cycling genes [7], 232 were detected in our grouped analysis, including *per*,*tim*, *vri*, *Pdp1*, and *cry*. The *Clk* gene was newly detected with easyClock in the grouped analysis (Fig. 4F), highlighting the tool’s high sensitivity for detecting low-amplitude cycling patterns (Fig. 4G). Overall, easyClock offers an efficient, user-friendly platform for rapid analysis of time-series omics data.

## Conclusions

Over the past several decades, various analytical and statistical methods have been developed and adopted in chronobiology, ranging from visual inspection of actograms to sophisticated mathematical statistics [28]. Among these, Analysis of Variance (ANOVA) has served as a fundamental tool for detecting the oscillatory characteristics of circadian data. However, ANOVA alone cannot directly demonstrate the presence of rhythmicity. To overcome this limitation, model-fitting techniques have been introduced. In response to the need for a versatile, accessible, and user-friendly circadian analysis toolkit, we developed easyClock—a GUI application that integrates comprehensive fitting models for circadian rhythm detection. Compared to other tools such as Python packages with GUIs (e.g., pyBOAT [29]) or web-based analyzers (e.g., BioDare2 [30]), easyClock not only integrates their advantages but also implements specific modules that are particularly helpful for chronobiologists, including noise handling, group comparisons, and editable visualizations (Table 2).

In easyClock, amplitude serves as a primary measure of rhythmic strength—a key concept in chronobiology that distinguishes robust circadian patterns from weak or noisy signals. Weak rhythms are typically characterized by irregular peaks, inconsistent phases, or diminished amplitudes. In addition to amplitude-based assessments, the chi-square periodogram (CSP) is commonly employed to quantify rhythmic strength by evaluating deviations from the global mean at each circadian time point [31]. Although high CSP values generally indicate strong rhythmicity, this method can be susceptible to false positives in the presence of substantial fluctuations within the time series [32]. As an extensible platform, future updates of easyClock intend to integrate more comprehensive and reliable methods for assessing rhythmic strength.

## Supplementary Information

Below is the link to the electronic supplementary material.

Supplementary Material 1

## Data Availability

The source code for the latest version of easyClock is available at https://github.com/HungryFly/easyClock_for_CircadianRhythms. To download the installation package and access version updates, please refer to https://github.com/Dr-WUBINBIN/easyClock/releases. Supplementary data are deposited in Zenodo (10.5281/zenodo.17179424). The transcriptomic dataset is provided in S2 Table of the published study (10.1371/journal.pgen.1009790). Project name: easyClock. Project home page: https://github.com/Dr-WUBINBIN/easyClock. Operating system: macOS and Windows. Programming language: Python. Other requirements: Installation packages (.dmg or.exe) can be installed on macOS (equipped with M1 or later chips) or Windows machines. License: GPL-3.0 open-source license. Any restrictions to use by non-academics: no restrictions.
