# Supplementary material for: easyClock: a user-friendly desktop application for circadian rhythm analysis and visualization
Source: BMC Bioinformatics. 2025 Dec 5;27:7. doi: 10.1186/s12859-025-06340-9 (PMC12797594; doi:10.1186/s12859-025-06340-9)
Supplement: Supplementary file 1 — Supplementary Material 1 [file 12859_2025_6340_MOESM1_ESM.docx]

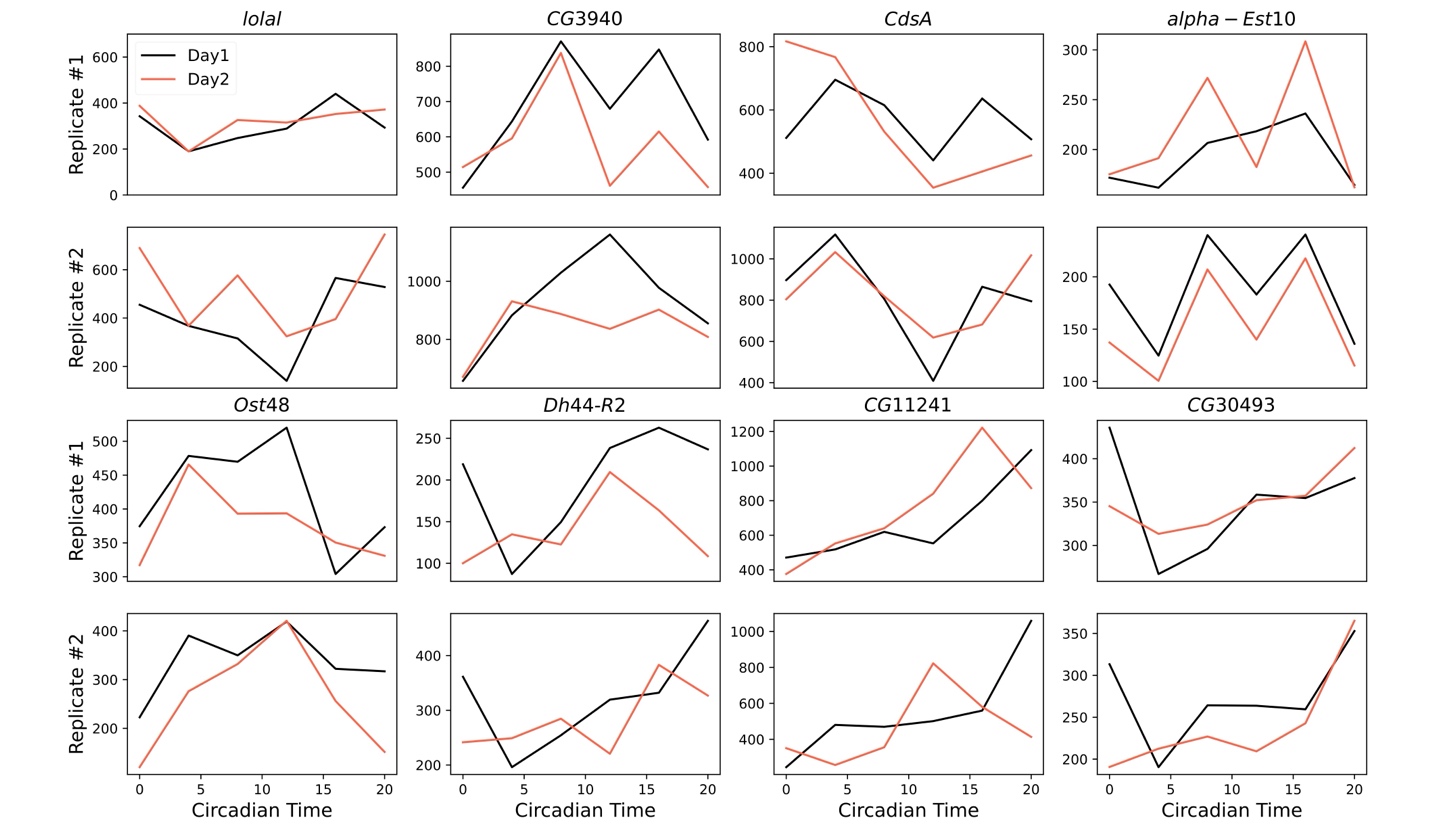


**Supplemental Figure 1: Newly identified cycling genes using the hybrid analysis approach with easyClock.**

Relative expression levels of eight genes across two days in two replicates. Day 1 and 2 are represented by black and red lines. Positive correlations between two replicates were determined by Spearman correlation. All expression data are from the transcriptomic dataset of You et al.
